# Prenatal and Postnatal Exposure to DDT by Breast Milk Analysis in Canary Islands

**DOI:** 10.1371/journal.pone.0083831

**Published:** 2014-01-08

**Authors:** Oriol Vall, Mario Gomez-Culebras, Carme Puig, Ernesto Rodriguez-Carrasco, Arelis Gomez Baltazar, Lizzeth Canchucaja, Xavier Joya, Oscar Garcia-Algar

**Affiliations:** 1 Unitat de Recerca Infància i Entorn (URIE), Institut Hospital del Mar d'Investigacions Mèdiques (IMIM), Barcelona, Spain; 2 Red de Salud Materno-Infantil y del Desarrollo (SAMID), Instituto Carlos III, Madrid, Spain; 3 Departament de Pediatria, Obstetricia, Ginecologia i Medicina Preventiva, Universitat Autònoma de Barcelona, Barcelona, Spain; 4 Departamento de Cirugía Pediátrica, Hospital de la Candelaria, Universidad de Tenerife, Santa Cruz de Tenerife, Spain; University of Missouri, United States of America

## Abstract

**Introduction:**

The use of *p,p′*-dichlorodiphenyltrichloroethane (DDT) has been banned since the late 1970s due to its toxicity. However, its long half-life makes it persistent in the environment and, consequently, almost everyone has DDT residues in the body. Human milk constitutes an ideal non-conventional matrix to investigate environmental chronic exposure to organochlorine compounds (OCs) residues. The study aimed to identify potential population risk factors of exposure to DDT due to the proximity to countries where it is still used.

**Methods:**

Seventy-two consecutive lactating women were prospectively included in Tenerife, Canary Islands (Spain). A validated questionnaire was used to obtain socioeconomic, demographics data, and daily habits during pregnancy. DDT levels in breast milk were measured by gas chromatography with-electron capture detector (GC-ECD). Anthropometrics measurements in newborns were obtained.

**Results:**

Thirty-four out of 72 (47.2%) of the analysed milk samples presented detectable levels of DDT (mean: 0.92 ng/g), ranging between 0.08 to 16.96 ng/g. The socio-demographic variables did not significantly differ between detectable DDT and non-detectable DDT groups. We found positive association between DDT levels and vegetables (OR (95%CI): 1.23 (1.01–1.50)) and poultry meat (OR (95%CI): 2.05 (1.16–3.60)) consumption, and also between the presence of DDT in breast milk and gestational age (OR (95%CI): 0.59 (0.40–0.90)).

**Conclusions:**

DDT is present in breast milk of women at the time of delivery. Residual levels and the spread from countries still using DDT explain DDT detection from vegetables and from animal origin food. The presence of this compound in breast milk represents a pre- and postnatal exposure hazard for foetuses and infants due to chronic bioaccumulation and poor elimination, with possible deleterious effects on health. This data should be used to raise awareness of the risks of OCs exposure and to help establish health policies in order to avoid its use worldwide and thus, to prevent its propagation.

## Introduction

According to the Stockholm Convention, Organochlorine Compounds (OCs) are considered Persistent Organic Pollutants (POPs). Their production and usage is forbidden in many countries due to toxicity, persistence, mobility and bioaccumulation in the environment. Due to their high lipophilicity and persistency they can bioaccumulate through the food chain [1], [2]. Since the discovery of its insecticidal properties in 1939, dichlorodiphenyltrichloroethane (DDT) was used extensively all over the world as a domestic and agricultural pesticide.

However, in Spain, as in most European countries, DDT was banned in the late 70 s [3], [4]. Currently, it is still used in African countries, such as Morocco [5].

The Canary Islands are located in the Atlantic Ocean, only 100 km away from the North African coast (South-West of Morocco). This proximity facilitates OCs transport by air and water, becoming sources of propagation. The economy of Canary Islands is based mainly on tourism and, to a much lesser extent, on farming and fishing. In the last decades farming in these Islands has turned into an intensive type of agriculture (plastic greenhouses) [6]. It is well known that intensive agriculture uses pesticides in large amounts [7].According to calculations, in 2001 the Canary Islands consumed 12 times more pesticides by hectare than the rest of Spain [8]. Previous data confirms that the general population in the Canary Islands presents with dichlorodiphenyldichloroethylene (DDE), the main metabolite of DDT [6].

Except for the individuals exposed through their occupation, most of the exposure to these compounds occurs through diet, especially food with high content of fat [9] such as meat, fish, poultry and dairy products. In the organism, due to their lipophilic character, POPs accumulate in the adipose tissue. During the production of breast milk, the human body uses lipids from the adipose tissue, and subsequently the accumulated POPs from the adipose tissue can migrate to breast milk [10]. Therefore, while providing the necessary nutrients for the correct development of the infant, human milk is also a source of lipophilic environmental pollutants [10]–[13].

The purpose of this study was to determine the levels of DDT in breast milk from a group of lactating mothers from the Canary Islands, and ascertain possible associations between this exposure and socio-demographic coordinates, diet and mother's habits, and their potential impact on the newborn's health.

## Materials and Methods

### Subjects and study design

A cross sectional descriptive pilot study was conducted in Tenerife, the largest and most populated island of the seven islands that make up the archipelago of the Canary Islands (Spain). The economy of Tenerife is based on a few sectors: tourism (78%) industrial activities (9%) and, to a lesser extent, farming and fishing (2%). Agriculture is centred on the northern slopes. In the last decades, farming in these Islands has turned to an intensive kind of agriculture (plastic greenhouses).

The study was carried out in the Paediatric Department of the Hospital La Candelaria of Santa Cruz de Tenerife, Spain, the main hospital of the island. A total of 72 consecutive pregnant women agreed to participate in the study and signed an informed consent form. The inclusion criteria were to be pregnant, to accept enrolment in the study and to sign a mother–newborn dyad informed consent. The exclusion criteria were not to sign the informed consent and if a breast milk sample was not obtained or was inadequate for DDT analysis. The study was approved by the local Ethics Committee (Comité de Ética e Investigación Clínica (CEIC), Hospital la Candelaria, Santa Cruz de Tenerife) in accordance with the Helsinki Declaration. The enrolment period was between August 2006 and June 2007.

### Determination and quantification of DDT in breast milk samples

On the day of delivery, breast milk was collected and stored at −20°C until analysis. A simple, sensitive and efficient liquid-liquid extraction method prior to analytical detection was used in this study with minor modifications [14]. PCB#1 and PCB#209 were added as internal standards to 200 µL of breast milk sample previously homogenized by vortexing it. The breast milk sample was homogenized with anhydrous sodium sulphate and extracted with n-hexane. At this point 1 µL of the organic layer was injected into the gas chromatograph with flame ionisation detector (GC-FID) in order to determine the concentration of fatty acid methyl esters (FAMEs). Then, the sample was extracted with 1 ml H_2_SO_4_ (96%) three times, mixing and centrifuging each time. Analyses of OC were carried out in a gas chromatograph with electron capture detection (GC-ECD) equipped with a fused silica, 5% diphenyl–95% dimethylsilicone (SGE, Ringwood, Australia) open tubular column of 25 m, 0.53 mm I.D. and 0.25 µm film thickness. The initial temperature of the oven was 140°C, raised to 267°C at a rate of 2.5°C/min. Injector and detector temperature were 290°C and 310°C, respectively. Carrier gas (He) was set at a linear velocity of 25 cm/s. Make-up gas (N_2_) was adjusted to a flow of 60 ml/min. Quantification of the OC compounds was made using the PCB#209 as internal standard. The concentration of DDT in breast milk is expressed in ng/g. The limit of detection (LOD) and limit of quantification (LOQ) were around 1 ng/g [15].

### Study variables

A previously validated questionnaire [16] was administered the day after delivery by trained interviewers and was focused on socio-demographic, dietary and lifestyle information, with the following variables: age, socio-economic status, area of residence, country of origin, length of time living on the island, educational level, parity, working status, tobacco and alcohol consumption. . The dietary information regarding the period of pregnancy was collected using a food frequency questionnaire of 16 items. Participants were asked how many times per week, on average, had consumed each item during the last month of pregnancy. Also, they were asked about the origin of their drinking water. Obstetric history of the mother was collected and neonatal anthropometric characteristics using customized growth charts for birth weight and height and clinical examination at birth were also recorded.

### Statistical analysis

Descriptive statistics of DDT skewed distribution in breast milk were performed using mean, median, and percentiles. The dependent variable was defined as dichotomous DDT level (detected vs non-detected). Preliminary association between socio-demographic, life style characteristics of pregnant women and neonatal outcomes with foetal exposure to DDT were done by Student's t test for continuous variables and Chi-square test for dichotomous variables. Multivariate logistic regression was adjusted for maternal age and gestational age and was used to find positive associations to DDT.. Potential determinants were analysed using dichotomous DDT levels, taking into account confounding variables. Smoking was included as a potential confounder. Statistical significance was set at p<0.05. Database management and statistical analysis were performed with SPSS v 20.0 (SPSS, Chicago, IL, USA).

## Results

Seventy two breast milk samples (participation rate: 49.65%) were used to determine the presence of DDT. DDT was detected in 34 breast milk samples (47.22%). The mean concentration (SD) was 0.92 (2.40) ng/g, ranging between 0.08 to 16.96 ng/g. The percentile 25^th^–75^th^ ranged between 0.00–1.19 ng/g. The distribution of parental socio-demographic characteristics in two categories (DDT detected and DDT non-detected) is shown in **Table 1**. More than 80% of mothers with detectable levels of DDT in breast milk were Spanish with an average age of 30.48 (5.31) years. The area of residence, length of time living on the island and maternal profession had no influence on breast milk values of DDT.

**Table 1:** Parental socio-demographics characteristics by prenatal and postnatal exposure to DDT detected in breast milk.

		Breast milk positive samples to DDT (n = 34)	Breast milk negative samples to DDT (n = 38)	*p-value*
**Maternal age** (years), mean (SD)		30.59 (5.83)	30.37 (4.85)	0.865
**Parental country of origin** (Spain/Other) (%)				
	Non-Spanish mothers	20.6	10.5	0.236
	Non-Spanish fathers	21.2	15.8	0.556
**Parental Educational Level** (%)				
Mother's	Non finalized elementary school studies	12.5	21.6	0.489
	Elementary school studies	40.6	43.2	
	University Studies	46.9	35.1	
Father's	Non finalized elementary school studies	21.9	33.3	0.568
	Elementary school studies	78.8	84.2	
	University Studies	21.2	15.8	
**Parental employment** (yes) (%)				
Unemployed mother		23.5	21.6	0.848
Unemployed father		0	2.6	1
**Parental socioeconomic status (%)**				
Mother's	Skilled	46.2	65.5	0.148
	Unskilled	53.8	34.5	
Father's	Skilled	58.6	51.4	0.556
	Unskilled	41.4	48.6	
**Area of residence (%)**				
	Rural (<10.000 inhab.)	17.6	15.8	0.975
	Semi-rural (10–100.000 inhab.)	26.5	26.3	
	Urban (>100.000) inhab.)	55.9	57.9	

Chi-square test; p<0.05.

Anthropometrics characteristics and perinatal history data are shown in **Table 2**. The mean gestational age was 39.2 (1.59) weeks. Newborns from mothers with detectable levels of DDT presented a significantly lower gestational age in comparison to the newborns of mothers with non-detectable DDT levels (p = 0.002). The presence of DDT in breast milk showed a significant negative correlation with the newborn's gestation time. (Spearman's rho: −0.373; p = 0.001). Women with detectable levels of DDT had an 8.8% of premature birth, compared with 2.7% for the mothers with no detectable DDT levels. (p = 0.344). Weight, length and cranial perimeter at birth did not differ between the groups exposed or non exposed to DDT. There were no differences between the two groups with respect to other conditions such as chromosomic alterations, increased risk of perinatal infection, hypoglycemia and/or congenital hip dysplasia.

**Table 2:** Obstetric and anthropometric characteristics of the newborns according to the results obtained.

	Breast milk positive samples to DDT (n = 34)	Breast milk negative samples to DDT (n = 38)	*p-value*
**Previous pregnancies** (%)			
0	47.1 (16)	55.3 (21)	0.244
1	20.6 (7)	28.9 (11)	
>2	32.4 (11)	15.8 (6)	
**Previous premature infants** (%)			
Yes	0	2.6 (1)	1
**Previous abortions** (%)			
Yes	35.3 (12)	18.9 (7)	0.119
**Children characteristics at birth**			
Gender; Female (%)	38.2 (13)	55.3 (21)	0.148
Gestational age (week), mean (SD)	38.4 (1.4)	39.4 (1.6)	0.008
Prematurity (%)	8.8 (3)	2.7 (1)	0.344
Weight at birth (g), mean (S.D.)	3335.29 (474.6)	3357.50 (466)	0.856
Length at birth (cm), mean (S.D.)	50.5 (2.3)	51.1 (3.2)	0.341
Cranial Perimeter (cm), mean (S.D.)	34.4 (1.4)	34.2 (14)	0.526
**Clinical diagnosis at birth** (yes), (%)			
Perinatal History	23.5 (8)	34.2 (13)	0.32
Chromosomic Alteration	0	0	NA
Loss of foetal well being	0	2.6 (1)	1
Risk of Perinatal Infection	8.8 (3)	23.7 (9)	0.091
Hypoglycemia	8.8 (3)	5.3 (02)	0.662
Developmental dysplasia of the hip	0	0	NA
Other outcomes	2.9 (1)	0	1

NA: Not applicable.

Chi-square test; p<0.05.

Maternal lifestyle, chemical exposure and dietary habits during pregnancy are presented in **Table 3**. Tobacco consumption before pregnancy was documented in 43.3% of the women positive to DDT in comparison to 31% with negative DDT result (p = 0.329). A frequent intake of vegetables was associated with detectable DDT levels in breast milk (p = 0.014). The presence of DDT in breast milk correlated with the average consumption of vegetables (Spearman's rho: 0.301; p = 0.013). Moreover, a frequent intake of poultry meat was also associated with detectable DDT levels in breast milk (p = 0.025). Nevertheless, the presence of DDT in breast milk did not correlate with the average consumption of poultry meat (Spearman's rho: 0.231; p = 0.058). Detectable DDT levels in breast milk were not associated with other categories of food and fish consumption.

**Table 3:** Maternal lifestyle, chemical exposure and dietary habits during pregnancy.

	Breast milk positive samples to DDT (n = 34)	Breast milk negative samples to DDT (n = 38)	*p-value*
**Maternal tobacco smoke exposure** (yes), (%)			
Smoking before pregnancy	43.3 (13)	31.0 (9)	0.329
Smoking during pregnancy	18.2 (6)	24.3(9)	0.532
**Drug abuse** (yes), (%)	3.0 (1)	5.4 (2)	0.240
**Alcohol consumption** (yes), (%)	0	0	NA
**Medicine use** (yes), (%)			
Antidepressants	10.0 (1)	5.7 (2)	0.284
Vitamin supplements	12.1 (4)	19.4 (7)	0.689
Antibiotics	21.2 (7)	16.7 (6)	0.233
**Water source (%)**			
Running Water	26.5 (9)	32.4 (12)	0.697
Private Well	2.9 (1)	0	
Mineral Water	70.6 (24)	67.6 (25)	
**Diet** (times per week), mean (SD)			
Full-Fat Milk	8.21 (9.2)	6.62 (8.40)	0.364
Fat-Free Milk	10.59 (11.21)	10.84 (10.59)	0.788
Fruit	8.65 (5.37)	8.89 (6.05)	0.807
Vegetables	7.50 (4.06)	6.30 (3.75)	0.014
Eggs	2.62 (1.65)	2.30 (1.45)	0.332
Butter and/or margarine	2.74 (2.68)	3.03 (2.64)	0.528
Legume	1.71 (1.19)	1.81 (1.15)	0.592
Nuts	1.24 (2.10)	0.62 (1.44)	0.282
Red meat	2.21 (2.56)	1.92 (2.25)	0.678
Processed meat	0.56 (0.89)	0.43 (0.83)	0.463
Poultry meat	2.65 (1.32)	2.0 (0.8)	0.025
Fish	1.62 (1.15)	1.57 (0.95)	0.905
Precooked food	0.35 ( 0.64)	0.57 (1.04)	0.491
Canned food	1.91 (1.84)	1.76 (1.64)	0.868
Commercial juice	4.59 (4.62)	6.3 (5.74)	0.217
Soft drinks	2.05 (3.69)	4.7 (4.0)	0.624

NA: Not applicable.

Chi-square test; p<0.05.

Multiple linear regression was performed but we didn't obtain any significance probably due the small size of the sample. **Table 4** shows the OR and 95%CI obtained in the adjusted logistic regression model. This model includes only the statistically significant variables from the univariate logistic regression model. The model was adjusted using confounding variables such smoking, maternal age or newborn gender. The gestational age (OR (95%CI): 0.59 (0.40–0.90)), the intake of vegetables (OR (95%CI): 1.23 (1.01–1.50)) and poultry meat (OR (95%CI): 2.05 (1.16–3.60)) were significantly associated with DDT detection in breast milk.

**Table 4:** Multivariate logistic regression model that associates gestational age, vegetables and poultry meat consumption with the presence of DDT in breast milk.

Variable	OR Adj	CI (95%)	*p-value*
Gestational age	0.598	0.397–0.900	0.014
Vegetables	1.230	1.007–1.502	0.042
Poultry meat	2.045	1.161–3.604	0.013

Adj: Adjusted for maternal age.

Calibration of the model (Hosmer & Lemeshow test) p = 0.161.

Discrimination power (AUC): 0.803 (0.693–0.912).

## Discussion

This study found detectable levels of DDT in 34 (47.2%) of 72 breast milk samples obtained from a population of lactating women from Tenerife (Canary Islands). Our data confirms that almost half of the pregnant women from our study performed in the Canary Islands, present unexpected DDT residues in breast milk. We found that the concentrations of DDT observed in the present study were within the range reported by other studies performed in similar areas [17]–[20]. However, DDT levels documented in this study were considerably lower than the levels found in human breast milk from China, South-Africa and other Mediterranean countries [21]–[27]. This variability observed in DDT concentration between different countries could be explained in part because some of them still produce and/or use DDT. This fact represents a source of propagation by air, water and exportation of products for human consumption. However, there is evidence that POPs concentrations have been decreasing significantly in the last four years due to bio-monitoring program interventions [28].

Taking into account that one of the main sources of DDT is the environment, we studied the possible differences in DDT levels associated with differences in urbanization of our population. In our study, DDT levels were not different in women living in urban or rural zones. This data differs from other studies in the same area. Zumbado M *et al.* found high levels of DDT in Tenerife and Gran Canaria Island (with many more inhabitants, with the largest urban areas and the largest surface covered by plastic greenhouses ) probably due to higher intake of dairy products and of lipids and saturated fatty acids [6], [29].

It has been shown previously that DDT levels increased proportionally with age since OCs accumulate in adipose tissue over time [6], [30], [31]. We didn't find an association between maternal age and accumulation of DDT in breast milk. This fact can also be related to the so-called “cohort effect”, because older subjects, born before restriction of the production and use of DDTs, would start with a higher body burden of POPs in comparison to younger people [31]–[34]. With respect to the perinatal effects of DDT exposure, it is well known that this pollutant is associated with negative reproductive outcomes in human studies [35], [36]. We found a significant negative correlation between gestational age and DDT levels. This observation was in accordance with other authors that studied the exposure to POPs during prenatal life [37], [38]. However, we didn't find any association between DDT exposure and birth outcomes such birth weight [39], [40].

Food is considered a constant source of exposure; Schafer *et al.* claimed that every food group usually contained at least five OCs, with DDT and dieldrin being the most frequent ones. [41]. Although ingestion of legumes, vegetables and bread was related to lower serum and adipose tissue concentrations of DDT [31], our data showed a statistically significant correlation between DDT levels and vegetables consumption. DDT residues in soil due to its use in past decades and ground water pollution in the islands can explain the contamination. Canary Islands, like all vulcanic islands, have geological characteristics that facilitate soil and ground water contamination (low organic matter). This type of soil and water saturation by pesticides has also been shown in the Hawaii Islands [42]. This feature, plus the proximity of the islands to Africa and the high consumption of vegetables imported from countries where these compounds are still used, may explain the presence of some DDT residues in lactating women eating vegetables [32], [43].

Poultry meat seems to be another intake source of DDT in our study. The importance of eggs, chicken and meat consumption as a source of OCP has been established worldwide [44]–[46]. Chicken meat is among the most popular food items in the diet of many communities and because of its high fat content it increased our concern related to human exposure to OCPs. Animals intended for human food may absorb pesticides from residues in their feed, water or during direct/indirect exposure in the course of pest control. The literature indicated that poultry feed could be one of the major sources of contamination through meat and eggs [47]. In order to minimize intake of OCP residues, people should adapt their pattern of food consumption (including a limitation of the consumption or including different types of meat).

## Limitations

The moderate participation rate (49.65%) and subjects selected from a single hospital, in spite of the fact that Hospital La Candelaria in Tenerife has the largest number of births on the island, precludes generalization of the results to the study area population as a whole. , Being only a preliminary study, . we can confirm a relationship between vegetables consumption and higher risk of high DDT levels, however increasing the sample size could provide results which using multivariate analysis could potentially determine personal characteristics associated with higher risk for elevated DDT levels. Additional data in dietary habits questionnaire are required to establish a relationship between diet patterns and positivity for DDT and concerning effects of foetal exposure to DDT. Another factor to be considered is that our dietary habits questionnaire shows the consumption only in the last month and in some cases, the food habits reported by the women may not have been representative for the long term food habits. . Several studies in Spain did not find significant associations between levels of these pesticides and dietary patterns [48]. DDT levels in food can indicate its recent use in agricultural areas. Nevertheless, due to the obvious benefits of eating vegetables and fruits for proper foetal development, caution should be exercised when making sweeping dietary recommendations for pregnant women.

Ongoing use of DDT for malaria prevention, its environmental persistence, documented adverse reproductive effects in animals, and inconsistent findings across human studies, justifies continued exploration of the DDT and DDE impact on human beings. The results of this study highlight the importance of carrying out similar studies in this field.

## Conclusion

Despite DDT being banned in Spain since 1977, Canary Islands population is still exposed to this insecticide. It is important to emphasize that with one isolated exception, concentrations were under the acceptable criteria by WHO, suggesting that infants in the island are exposed to small quantities of DDT [49]. However, even after controlling for smoking, there was still a positive association of DDT and gestational age. There is evidence that POPs concentrations have decreased significantly, but, because of globalization, children eat a variety of vegetables from all over the world, including countries in which usage of DDT has not been restricted yet, due to its usefulness in eradicating diseases such as malaria. Virtually, all populations worldwide bear a body burden of POPs with large interindividual and inter-population differences [28], [49]. Therefore, studies are essential to establish reference concentrations, to analyze predictors of exposure, to increase public awareness, to stimulate more energetic policies and population strategies and, hence, to diminish the burden of exposure, especially in infants. The accumulation of these compounds in the fatty tissue over the mother's life may be an important source of exposure for children, both during gestation and through breastfeeding. DDT levels measured in our study were not associated with perinatal outcomes such as weight or length at birth. However, further research regarding other OCs pesticides and especially the chemicals currently used and their doses is needed, with the goal to improve knowledge and implement health and policy strategies in order to regulate their use and to prevent its propagation.
