# Correction: Prenatal and Postnatal Exposure to DDT by Breast Milk Analysis in Canary Islands

**DOI:** 10.1371/journal.pone.0199904

**Published:** 2018-06-25

**Authors:** Oriol Vall, Mario Gomez-Culebras, Carme Puig, Eva Rodriguez-Carrasco, Arelis Gomez Baltazar, Lizzeth Canchucaja, Xavier Joya, Oscar Garcia-Algar

The fourth author’s name is spelled incorrectly. The correct name is: Eva Rodriguez-Carrasco.
